# Selenium in Camel – A Review

**DOI:** 10.3390/nu1010030

**Published:** 2009-07-08

**Authors:** Bernard Faye, Rabiha Seboussi

**Affiliations:** 1 Département Environnement et Sociétés, CIRAD, Campus International de Baillarguet, UR 18, TA/C Dir B, 34398 Montpellier cedex, France; 2 5902 Roi René, Anjou, QC H1K3E7 Montréal, Canada; Email: rseboussi@yahoo.com

**Keywords:** selenium, camel, requirements, deficiency, toxicity, excretion

## Abstract

Requirements for trace minerals in camels, particularly selenium, are not well-known. Selenium supplementation using a pharmaceutical form or commercial mineral mixture is common practice in camels to address the cardiomyopathy often attributed to selenium deficiency. This supplementation is often empirical and based on estimated needs for cattle. Nowadays the use of selenium in animal foodstuffs is commonplace and further investigation of its metabolism (ingestion, dynamic of storage-destocking, excretion) in camels is warranted. The present review aimed to synthesize all the experimental research (comparative selenium status in cow and camel, response to different levels of supplementation at different physiological stages, excretion maternal transfer, experimental toxicosis) and field observations (deficiency, supplementation practices) undertaken in camels. The results underline the particularity of the unique metabolic profile of the camel and lead to practical recommendations for supplementation in camels, highlighting its relative sensitivity to excess Se intake at lower levels than in cattle. The maximal tolerable dose is 8 mg and the recommended doses range from 2 to 4 mg.

## 1. Introduction

Camels have some physiological peculiarities in their trace element metabolism due to their adaptation to arid conditions and poor mineral feeding resources [1,2]. Some studies concerning trace elements such as copper, zinc, iron, manganese in camel have shown specific responses of this species to mineral supplementation or deficiencies. In general, the camel metabolism seems to anticipate mineral under-nutrition periods of its life with different mechanisms: increase of the absorption capacity in scarcity periods (copper, zinc) [3], higher storage capacity (copper) [4], tolerance for minerals and electrolytes in excess (calcium, phosphorus, sodium) [5,6], maintenance of enzymatic activity in deficient period (caeruloplasmin, superoxide-dismutase) [7,8].

The adaptation to desert life means an addition of small metabolic improvements which provide no comparative advantage when they are considered one by one, but give a full meaning to the reputation of this species when they are considered as a whole. This probably explains why the camel is able to survive under desert conditions. 

Concerning selenium, there is little evidence to date of clinical deficiencies or toxicities, and up to recently, few available data on selenium requirements and metabolism in this species. However, new findings on selenium metabolism in dromedary camel have been recently reported [9,10,11,12,13]. The present study aims at giving a progress report on current knowledge concerning the status of selenium and its metabolism in the dromedary based on all data available in the scientific literature.

## 2. Normal Selenium Level in Camel Blood

The mean concentration of blood/serum selenium reported in the literature for large animals was around 100 ng/mL, a value considered as sufficient for the maintenance of suitable metabolic functions [14]. In the dromedary from Morocco, Hamliri *et al.* [15] observed in whole blood values between 109.1 and 117.8 ng/mL, varying according to age and sex, being thus similar to those reported in sheep in the same area. Similar figures were recorded by Liu *et al.* [16] in China, with concentrations varying from 97 to 112 ng/mL. In Sudan, Abdel Rahim [17] reported values in whole blood varying between 25 and 53 ng/mL. Without specifying if it was whole blood or serum, Ma [18] reported higher values: 274 to 288 ng/mL. The analytical method used could also explain the observed differences.

Serum concentrations approached these last figures: 281 ng/mL on average in sera coming from the Sultanate of Oman (Faye, unpublished data). In Morocco, in dromedaries receiving probably a low Se basal diet, the plasma selenium concentration was quite lower, about 21 ng/mL [19]. In male adult camels in healthy conditions from Iran, the selenium concentration reported in serum was 12.6 ng/mL only [20]. In Saudi Arabia, serum Se values reported in young camels at the slaughterhouse varied between 5.3 and 131 ng/mL with 30% of samples higher than 100 ng/mL [21]. In the United Arab Emirates (UAE), the mean value was 200 ± 90 ng/mL in animals with no Se supplementation [22]. In recent experiments with different levels of Se supplementation, selenium content in serum for non-supplemented animals was on average 137.6 ± 18.7 ng/mL in non-pregnant, non-lactating camels [9] (Seboussi *et al.*, 2008), 109.3 ± 33.1 ng/mL in pregnant females, and 103.4 ± 28.7 ng/mL at milking period [11]. In small camelids such as llama [23], the selenium concentration in serum ranged on average between 213 and 203 ng/mL depending on the physiological status.

The variability was thus high and the range between 12 and 200 ng/mL with an average of 100 ng/mL. However, in most of the reported values, the selenium status of the diet was unknown even if Se supplementation was not distributed to the animals. Also, the analytical procedures were not described in all the cases and could differ between authors.

## 3. Selenium Deficiency

**Figure 1:**
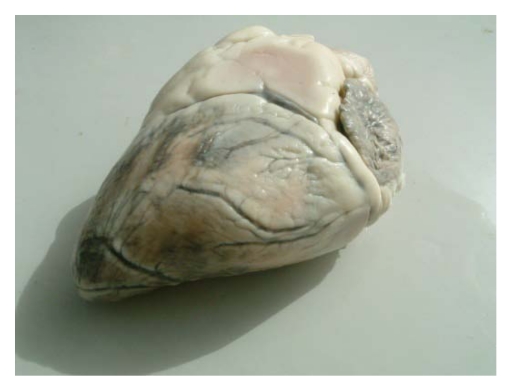
Degenerative myocarditis lesions in the heart of a one-month old camel calf (*Photo: R. Seboussi*).

For a long time, selenium deficiency has been suspected to occur in camels kept in zoological parks affected by cardiopathy or myopathy [24,25,26], but no clinical descriptions and laboratory analysis have been made in these reports to confirm the role of selenium. Also, in China Liu *et al.*  [16] suspected selenium deficiency in cases of sway-back in Bactrian camel. However, selenium deficiency with characteristic clinical signs has been recently reported. Selenium deficiencies affect generally young animals and are responsible for white muscle disease. The most important lesions are degenerative myocarditis and discoloration of skeletal muscle. In the UAE, soils and feedstuffs are generally considered deficient in selenium, and many cases of degenerative myocarditis (Figure 1) are observed with histological lesions similar to those in cattle [22,27]. 

When the skeletal muscles are affected, symptoms vary from mild stiffness to obvious pain upon walking, to an inability to stand. Camel calves may tremble in pain when held in a standing position. When the problem occurs in newborns – they are born weak and unable to rise. Sudden exercise may trigger the condition in older camel calves. When the disease affects the heart, the animal shows signs similar to pneumonia, including difficult breathing, fever with an elevated heart and respiratory rates.

In sick animals, between 2 and 12 weeks old, remarkable signs of anemia were observed with reduction of hemoglobin concentration, slight decline of PCV% and total erythrocytes comparing to the normal levels reported in apparently healthy animals of the same age [27], but these findings were not observed in three seleno-deficient adult camels from Saudi Arabia [28]. The histopathological findings showed alterations of the cardiac tissue with focal areas of non-inflammatory coagulative necrosis. The necrotic areas showed swollen myocardial fibers with granular cytoplasm and loss of striation. This was accompanied by severe blood vessels congestion, edema and lymphocytic infiltration. Calcium salt deposition was observed all over the necrotic area and fibrous area also [27].

The serum selenium level in deficient camels was obviously very low: in camel calves, the average level of Se serum in diseased cases was below 35 ng/mL [27] (and between 0.8 and 3.7 ng/mL in 3-yr animals [28].

## 4. Effect of Se Supplementation on Se Status in Camel

**Figure 2:**
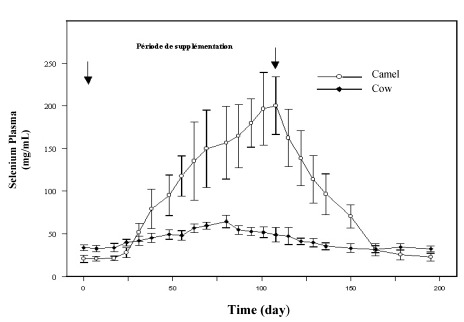
Comparative change in plasma selenium concentration in cow (●) and dromedary camel (○) receiving 2 mg/day selenium under sodium selenite form (reproduced from [19], with permission).

Few papers relate the impact of selenium complementation on the mineral status of camel and, generally, the doses applied for selenium deficiency control were those recommended for cattle. To our knowledge, the first trial achieved to assess the effect of selenium supplementation on the plasma selenium status was reported by Bengoumi *et al.*  [19]. These authors compared the selenium status of camels with that of cattle with similar weight and receiving daily 2 mg Se *per os* in sodium selenite form for two months. In this study, sharper increase of plasma selenium occurred in camels (10 times the plasma level before supplementation) compared to cows (twice the starting level) was observed (Figure 2). As the magnitude of the decrease of plasma selenium concentration after stopping supplementation was similar to the previous increase, it was supposed that plasma (or serum) selenium concentration in camel was an extremely sensitive indicator of selenium intake. The fast selenium depletion at the end of the supplementation period seemed also to indicate a better efficiency of selenium absorption and excretion in camel compared to cow.

In selenodeficient camels with muscular dystrophy, Al-Qarawi *et al.* [28] gave an oral treatment involving selenium – vitamin E (Bo-SE, Schering – Plough Animal Health, 2.19 mg sodium selenite + 50 mg vitamin E) by IM injection at a dose rate of 0.5 mg/kg body weight for two consecutive days. Following treatment, selenium concentration rose from on average 2.3 ng/mL up to 23.7 ng/mL, i.e., with a similar trend to that observed by Bengoumi *et al.* [19], who also observed that the selenium concentration was increased 10-fold after supplementation.

**Figure 3:**
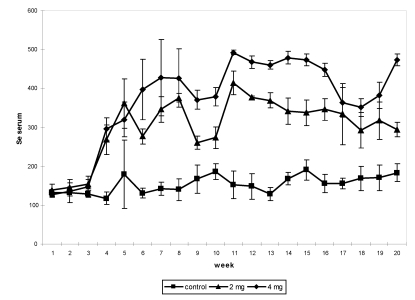
Change in serum Se (in ng/mL) concentration in non-pregnant and non-lactating camels according to the Se supplementation level: 0 (■), 2 (▲) and 4 (♦) mg daily by period*treatment (reproduced from [9], with permission).

In several studies on the effect of oral selenium supplementation [9,11,12,13,22], different levels of supplementation were tested up to the toxic limit, from 2 up to 16 mg/day under sodium selenite form. In the first experiment, 12 non-pregnant and non-lactating female camels shared into three groups received, after a two-week adaptation period, an oral Se supplementation (0, 2 and 4 mg, respectively) under sodium selenite form for three months. Feed intakes were assessed daily, blood samples and body weight were taken weekly, feces and urine samples were collected every two weeks up to one month after the end of the supplementation period. The Se concentration in serum had increased significantly in supplemented groups (Figure 3). The maximum level was observed in the period of supplementation in the camel receiving 4 mg (492.5 ng/mL), which was 4-fold compared to the value at the beginning of the trial (126 to 138.5 ng/mL depending on the groups).

**Figure 4:**
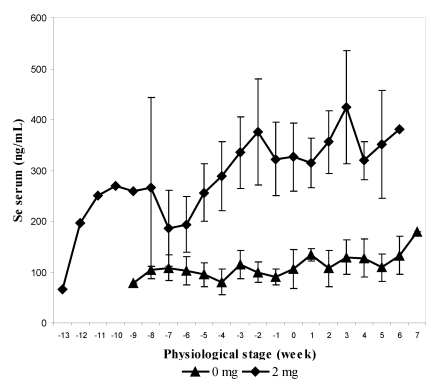
Biweekly changes (mean and S.E) in serum selenium concentration (in ng/mL) in camels before and after parturition according to the selenium supplementation level, 0 mg/day (▲) and 2 mg/day (♦) (reproduced from [11], with permission).

In the second experiment, 12 pregnant females, divided into two groups, received 0 and 2 mg Se respectively in sodium selenite form at the end of their gestation (last three months) and for up to one month at the beginning of their lactation. The supplementation was stopped after one month of lactation. As for the previous experiment, feces and urine samples were collected every two weeks. The mean value of selenium content in serum was significantly higher in supplemented group (2 mg) and was three-fold higher than the concentration compared to the control group (305.9 ± 103.3 ng/mL and 109.3 ± 33.1 ng/mL respectively). The maximum level was observed two weeks before calving in the group receiving 2 mg (638.7 ng/mL). The selenium level at parturition was still significantly higher in the treated group in spite of a slight decrease around the calving period (Figure 4). On average, serum Se concentration in control and treated groups was significantly higher after parturition (121.6 and 349.7 ng/mL, respectively) than before (97.4 and 272.7 ng/mL respectively) in spite of stopping supplementation in the treated group. 

**Figure 5:**
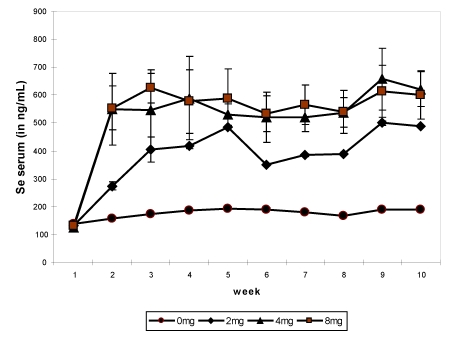
Weekly change in serum selenium according to Se supplementation in the basal diet of camels, 0 mg/day (●), 2 mg/day (♦), 4 mg/day (▲) and 8 mg/day (■) (reproduced from [13], with permission).

In the third experiment, eight young female camels shared into four groups of two 2-y old ones received a basal diet enriched with 0, 2, 4 and 8 mg selenium in sodium selenite form for 64 days. On average, the mean value of selenium in the serum was 176.3 ± 18.0 ng/mL in the control group, 382.7 ± 107.6 ng/mL in the group receiving 2 mg Se, 519.8 ± 168.4 ng/mL in the group receiving 4 mg Se and 533.4 ± 158.6 ng/mL in group receiving 8 mg Se daily. The weekly change showed a significant increase (*P <* 0.001) from week 2 up to the end of the experiment in the three supplemented groups compared to the control one (Figure 5). There was no difference between the groups receiving 4 and 8 mg Se. The maximum value (657.3 ng/mL) was observed in group 3 at week 9 and the minimum at the beginning of the experiment (124.1 ng/mL, also in group 3).

**Figure 6:**
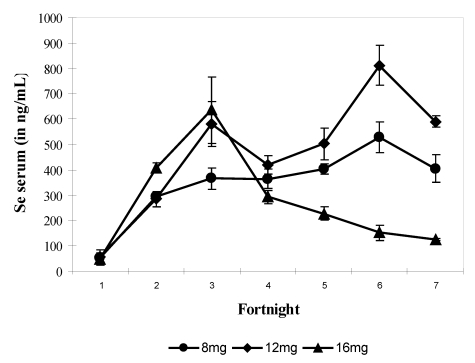
Changes in serum Se concentrations according to the selenium supplementation level in camels (Mean and S.E) at 8 (●), 12 (▲) and 16 mg/day (♦). The * points to the Se supplementation stopping in group 3 (reproduced from [11], with permission).

In the fourth experiment, the quantity of supplied selenium throughout the trial (90 days) was for each group of four camels, 2-year old, respectively 8 mg (i.e., 17.44 mg sodium selenite), 12 mg (i.e., 26.16 mg sodium selenite) and 16 mg (i.e., 34.88 mg sodium selenite) daily. Selenium supplementation was stopped immediately at the time of apparition of chronic selenosis and hepatoprotector was given to prevent death. Camels returned to normal good health gradually. On average the mean value of selenium in serum was 358.3 ± 210.8 ng/mL (n = 69) and varied between 16.3 and 899.8 ng/mL. The mean values of selenium in serum were 321.2 ± 140.5 ng/mL in group 1 (8 mg Se), 443.2 ± 231.1 ng/mL in group 2 (12 mg Se) and 298.04 ± 212.13 ng/mL in group 3 receiving 16 mg Se daily. The bi-weekly change showed a significant increase (P > 0.001) from fortnight 2 up to the end of the experiment for groups 1 and 2 and up to fortnight 3 for group 3 with a value of 767.15 ng/mL. Serum Se concentration decreased significantly in fortnight 4 in group 3 up to the end of the trial to reach a value of 129.86 ng/mL when Se supplementation was stopped when selenosis symptoms appeared (Figure 6). The maximum observed value was 899.87 ng/mL. 

**Figure 7:**
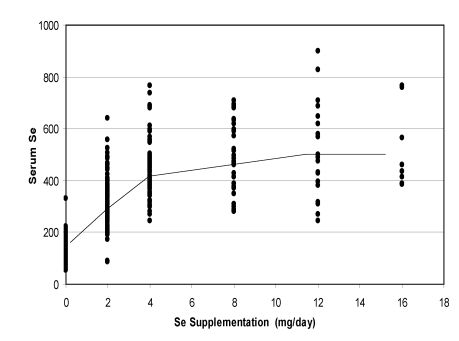
Change in camel serum selenium according to the level of oral supplementation (according to [9], [11][12] and [13]).

A meta-analysis of the data including oral supplementation from 0 to 16 mg/day (only values after at least one week of supplementation were taken into account) showed a clear linear relationships up to 4 mg, then a slight increase with a plateau after 12 mg/day (Figure 7).

## 5. Maternal Transfer of Se

In the previous experiment on lactating camels [11], Se was determined in camel calves after birth and in milk. Se supplementation involved dams only. Se serum concentrations in camel calves at parturition were 106.3 ± 26.5 and 273.2 ± 48.0 ng/mL, in the control (0 mg/day) and treated groups (2 mg/day), respectively. This significant difference (*P* < 0.001) was maintained for the entire milking period: 103.4 ± 28.7 and 248 ± 14.1 ng/mL in the control and treated groups, respectively.

**Figure 8:**
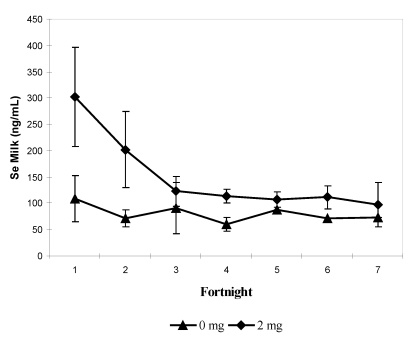
Biweekly changes (mean and S.E) in milk selenium concentration (in ng/mL) in she-camels for the three first months of lactation according to the selenium supplementation level: 0 mg/day (▲) and 2 mg/day (♦) (reproduced from [11], with permission).

In milk, the Se concentration varied from 39.5 to 482.6 ng/mL, with an average of 86.4 ± 39.1 ng/mL in the control group and 167.1 ± 97.3 ng/mL in the treated group. At birth, Se concentration in colostrum was three-fold higher in the treated group: mean value 302 ± 94.60 *vs* 108.2 ± 43.9 ng/mL (*P* < 0.001). In both groups, Se milk concentration decreased and after the second milk sampling, no significant difference was observed (Figure 8). By considering Se concentration in colostrum and the status of the mothers and of their camel calves at parturition, positive correlations were observed with serum Se in mothers (r = 0.659; *P* < 0.05) and in calves (r = 0.689; *P* < 0.05).

The reported values of selenium in camel milk are quite scarce. Al-Awadi and Srikunar [29] reported a much lower value (13.9 ± 2.4 ng/mL) than Seboussi *et al.* [11], but the former did not mention the lactation stage. In a meta-analysis performed on cattle’s data [30], it has been considered that the selenium increase in milk was on average 12.6 ng/mL only after oral Se supplementation at a dose of less than 3 mg/day under selenite form. In comparison, the apparent good efficiency of Se transfer in camel milk has to be confirmed.

## 6. Correlations of Selenium with GSH-Px Activity and Vitamin E

**Figure 9:**
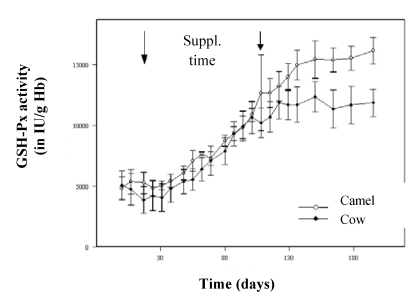
Comparative change in GSH-Px activity in cows (●) and dromedary camels (○) receiving 2 mg/day selenium under sodium selenite form (reproduced from [19], with permission).

For its transport in blood, selenium is linked to specific proteins (selenoproteins), including glutathione peroxidase (GSH-Px). In the comparative study of Bengoumi *et al.* [19], the increase of GSH-Px activity was similar in camels and cows for the supplementation period with a higher correlation in camels (r = 0.94) than in cows (r = 0.68). As for other species, GSH-Px is a good indicator of the Se status of camel. However, after the end of the supplementation, GSH-Px activity continued to increase in camels’ blood while it was stable in cows’ (Figure 9). A similar figure was observed by Seboussi *et al.* [9].

**Figure 10:**
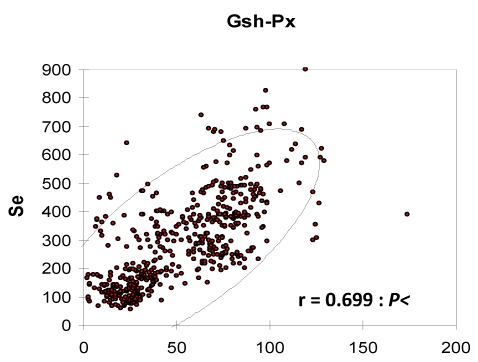
Relationship between Se serum and GSH-Px in camels according to data meta-analysis of [9], [11][12] and [13]).

This increase could be explained by the maintenance of the biosynthesis induction in the camel erythrocytes from the selenium probably stored in the erythrocytes, and a longer plasmatic half-life of GSH-Px compared to those of cattle. In fact, the erythrocyte GSH-Px activity being closely related to the half-life to the red blood cells, the enzymatic activity was higher in camels than in cows when selenium was depleted because of the longer survival of camel erythrocytes [31]. The linear relationship between erythrocyte GSH-Px and whole blood Se concentration was described in camesl by several authors [9,10,11,12,13,15,17,32] but with variable correlation coefficients (Figure 10).

Vitamin E is an essential component in the reproduction processes and performance of farm animals and acts in synergy with selenium (Se), especially in order to prevent white muscle disease (WMD) due to a severe deficiency. In the literature on camel from the UAE, the mean values were 1.13 ± 0.61 µg/mL (non-lactating and non pregnant), 1.12 ± 0.81 µg/mL (pregnant), 1.20 ± 0.80 µg/mL (lactating), 0.82 ± 1.06 µg/ml (new-born), 0.56 ± 0.22 µg/mL (young 2-y old) and 0.68 ± 0.36 µg/mL (Se intoxicated young camels) [9,10,11,12,13]. These results were quite similar to those described in young camels from Sudan (0.3 to 1.65 µg/mL) [33]. Similar results were reported by Al-Senaidy [34] and Mousa *et al.* [35]. In all the cases where serum Se and vitamin E were analyzed, no correlation was observed [10,11,12]. In case of Se intoxication, a tendency to the decrease of vitamin E in intoxicated animals with clinical signs was observed but no significant correlation was reported with serum Se concentration [10,12,13].

## 7. Se Excretion

**Figure 11:**
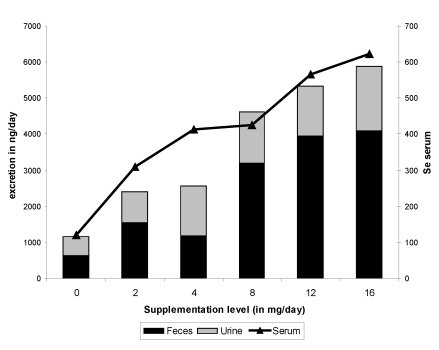
Fecal and urine excretion of selenium according to the level of supplementation (data meta-analysis of [9], [11][12] and [13]).

Very few data are available on fecal and urinary Se excretion in camel. According to the different trials reported above [9,11,12,13] with variable levels of Se supplementation in the diet, Se fecal excretion increased slowly up to 4 mg Se in the diet, then highly from 8 mg daily supplementation up to 16 mg (Figure 11). The total fecal excretion varied from 637.9 ng/day in non-supplemented camels up to 4,084.4 ng/day in camels receiving 16 mg Se/day in the diet. The total fecal excretion was comparable to urinary excretion when administering up to 4 mg supplementation, but the main part of Se excretion after 8 mg of supplementation was of fecal origin. The total urinary excretion varied from 518.5 ng/day (control groups) up to 1,795.9 ng/day (16 mg Se supplemented group). Forty-five percent of the excreted Se was from urine in non-supplemented animals *vs* 26-30% only in highly supplemented camels. Similar trends were observed with Se concentration in feces. Moreover, Se concentration in serum was highly correlated with Se concentration in urine and with fecal concentration and total Se fecal excretion but not with total urinary excretion (Figure 11). 

Similar change in Se excretion was observed in cattle [36]. When the dietary intake was increased from 0.15 to 0.40 mg/kg DM in cattle, the selenium concentration in feces and urine increased significantly from 370 to 780 ng/g DM and from 20 to 180 ng/mL, respectively [36], close to the results from Seboussi *et al.* [11]: on average 225 and 817 ng/g in the control and the treated group, respectively. But, contrary to this former observation achieved in dairy cattle, no linear effect was observed in camel.

The urinary selenium concentration is considered to be a more sensitive indicator of sodium selenite consumption than nutritional requirements [37]. Elsewhere, the camel is well-known for its water metabolism and its ability to excrete a more concentrate urine although the watering was *ad libitum* in the mentioned trials. The high Se urinary concentration, particularly in camels receiving Se in their diet, compared to cattle, seems to demonstrate a peculiar sensitivity to Se supplementation.

## 8. Se Storage in Organs

Selenium determination in organs has rarely been reported because it is of little clinical interest. In the wool of Bactrian camel from China, Liu *et al.* [16] reported values between 140 and 190 µg/kg, depending on their physiological status. Similar results have been published by Ma [18]: 190 to 210 µg/kg. These values corresponded to camels receiving 2 mg Se supplementation in the experiment of Faye and Seboussi [13] (163.6 µg/kg). In lambs, the wool Se concentration varied between 500 and 2,500 µg/kg, depending on the dietary Se level [38]. Part of the selenium ingested is involved in hair amino acids synthesis. It was suggested that a level of selenium content should be higher than 120 ppm in cow and calf hair to avoid nutritional myopathy. Season, color of hair, age and sex, affect the selenium content in hair. The selenium concentration was higher in winter than in summer and in dark color hair than in light color hair [39]. Hair appeared as the most sensitive organ to Se supplementation as it was reported on lamb [39] and cattle [40]. However, as with other minerals, the selenium concentration in hair is of limited interest [41].

**Figure 12:**
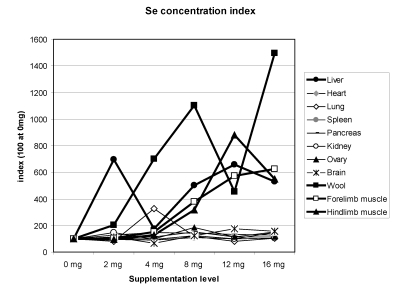
Selenium concentration index in the different camel organs according to the Se supplementation level in the diet. The index 100 corresponds to Se concentration for 0 mg supplementation (data meta-analysis of [9], [11][12] and [13]).

In the experiments of Faye and Seboussi [13], Seboussi *et al.* [9,11,12], and Seboussi [42], on average the highest total quantity of selenium was observed in the following order: in the liver (2727 µg), the kidney (807 µg), the lung (443 µg) and the heart (160 µg). Of course, a high quantity was also observed in muscle (2,513 µg). On average, whatever the Se supplementation level, the kidney (1,129 µg/kg), the liver (921 µg/kg), the hair (545 µg/kg), the forelimb muscle (421 µg/kg), the hind limb muscle (351 µg/kg) and the lung (308 µg/kg) had the highest Se concentrations. The total quantity was higher in supplemented groups but, except in hair, liver, kidney and muscle, the quantity was not clearly linked to the Se supplementation level. By giving an index of 100 for concentrations in animals receiving 0 mg Se supplementation, the main organs where selenium was stored were hair, liver and muscles, and to a lesser extent lung, ovary and pancreas (Figure 12). In Bactrian camel, one reference only was available for selenium concentration in organs [18]. In this study, kidney (3,100 to 3,900 µg/kg), liver, and heart (1,100 to 1,500 µg/kg), muscle and brain (620 to 640 µg/kg) were the organs with the highest Se concentrations. These values, except those of the liver, appeared much higher than those found by Seboussi *et al.* [11,12]. 

In the selenium tolerance trial carried out in lamb [38], the liver had the highest Se concentration (up to 2,000 µg/kg), followed by the kidney (around 1,000 µg/kg). In another study on sheep, selenium was found in the highest concentrations in the kidney, followed by the liver, pancreas, heart and skeletal muscle [41]. No linear trend of liver Se concentrations according to the Se supplementation level was observed in lamb [38]. In calves receiving 3 ppm dietary Se treatment, Se concentrations were 4,740 µg/kg in liver, 3,420 in kidney, 1,380 in heart and 340 in muscle [43]. Contrary to Cristaldi *et al.* [38], a regular increase of Se concentration with dietary Se level was observed by these authors. According to them, the kidney was the major organ involved in the storage of selenium at low Se supplementation, but at high intakes, the liver became the target organ [43,44]. Similar figures could be observed with camel [13].

However, considering the weight of whole carcass and of the different organs in camel, the total quantity of selenium in a camel of 200 kg carcass weight was around 100 mg with 90% in the muscle, 5.5% in the blood and 2.5% in the liver. Less than 1% was stored in the kidney.

## 9. Se Toxicity

At our knowledge, only experimental selenosis has been reported [10,13,43]. The first clinical disturbances appeared with a selenium supplementation of 8 mg/day. The first physiological symptoms were an increase of the respiratory rate, pulse rate, and internal temperature up to 40 °C.

**Figure 13:**
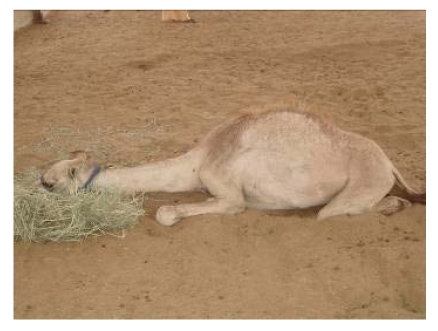
Camel in sternal decubitus position with neck extended on ground. (Seboussi, [42]).

**Figure 14:**
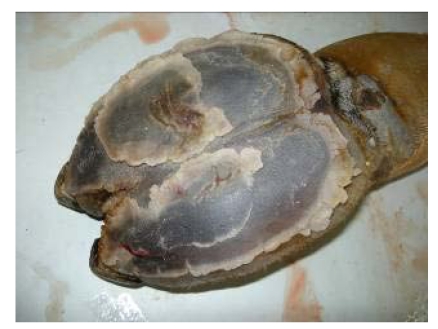
Fissured pads with necrosis on foot of camel receiving 16 mg Se/day (Seboussi, [42]).

The *clinical signs* occurred within two weeks, with hair discoloration, followed by alopecia, more severe in animals receiving a higher quantity of selenium (up to 16 mg/day). Enlargement of the inferior cervical lymph node was seen in all intoxicated animals**. **Camels tended to sit alone. Urinary excretion increased and dark watery diarrhea was also observed. Loss of appetite, thus loss of weight and weakness appeared. Tears with pale mucous were present as well as evidence of impaired vision. Dyspneic respiration and pain at auscultation appeared and camels adopted the sternal decubitus position and tended to rest their neck extended (Figure 13). Salivation occurred and finally camels showed no desire to eat and drink. The tail was elevated. Fissured pads appeared in all groups but more pronounced in groups receiving 12 and 16 mg. Consequently, camels had difficulty in walking (Figure 14). 

**Figure 15:**
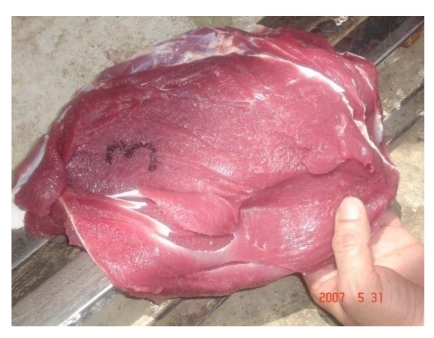
Discoloration of the red muscle in Se intoxicated camel receiving 16 mg Se daily (Seboussi, [42]).

After slaughter, intoxicated camels showed paleness in all abdominal muscles (Figure 15), paleness of diaphragm and intercostal muscles, hydrothorax, pulmonary emphysema. The texture of the liver and lung was not uniform. Heart, liver and kidney were congested and necrosed. 

**Figure 16:**
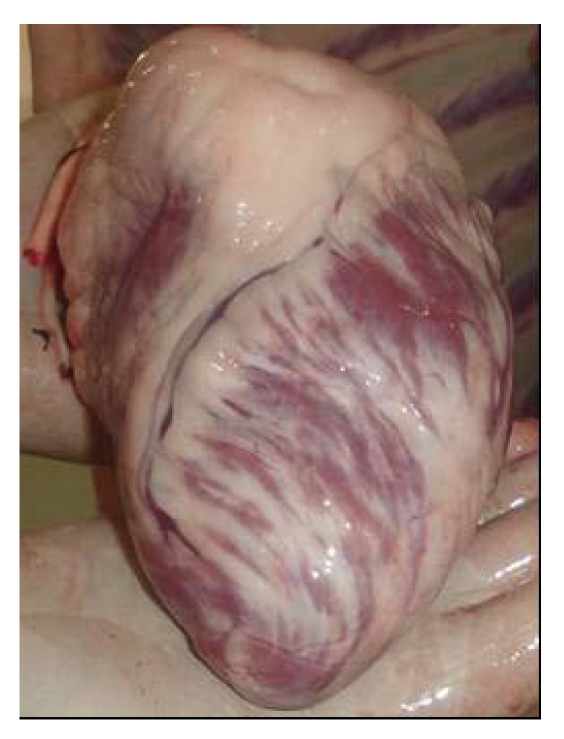
Heart discoloration and congestion in camel receiving 8 mg selenium per day for 45 days (Seboussi, [42]).

In addition to prior lesions, camels showed a flap heart with necrosis and congestion (Figure 16). However, the heart was partially white (fibrosis), congested and necrosed in camel receiving 8 mg. Hepatomegaly was observed in all animals, while pancreas was atrophied. Brain edema was also observed. 

**Figure 17:**
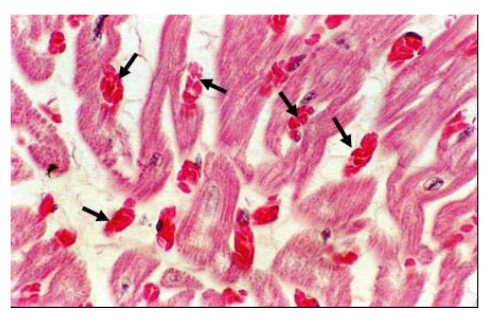
Congestion of the capillary vessels in the camel heart in camel receiving 12 mg Se/day (Seboussi, [42]).

**Figure 18:**
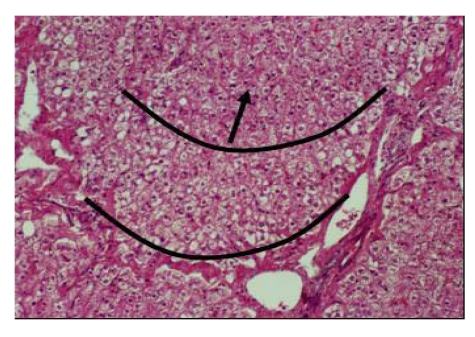
Hepatic cells degenerescence around the portal vein in camel receiving 16 mg Se/day (Seboussi, [42]).

*Histopathology lesions* involved all the organs, and the lesions increased with the quantity of selenium in the diet. Kidneys showed eosinophilic granulated material in diluted Bowman's space and convoluted tubules in addition to degenerative changes in epithelial lining cells. The heart showed proliferation of Purkinje fibers, capillaries congestion in Purkinje fiber tissues and sub-endocardial tissues, degenerative changes in myofibers. The cardiac tissues showed edematous fluid between more eosinophilic thick myocardial fibers (Figure 17). Vacuolar degenerative changes were observed all over the hepatic cells of the hepatic lobules, as well as congestion in central hepatic vein and hepatic sinusoids (Figure 18). In addition, focal areas of muscular hyalinization (non-inflammatory) and edema were observed in intercostal and diaphragm muscles. In addition focal coagulative necrosis areas appeared in pancreatic acinis. Hyaline degeneration of myofibers and edema was also observed in shoulder and intercostal muscles. The brain showed perivascular edema.

The clinical symptoms observed in camel were in accordance with previous signs reported in chronic poisoning in other species [45,46]. When selenosis injury occurred, the selenium accumulated mainly in the circulatory and respiratory system as well as in the organs of elimination [47]. These findings were in accordance with lesions observed in the heart, lung, liver, kidney and urinary bladder. After the liver, the kidney, particularly the cortex, retained the highest concentration followed by the glandular tissues, especially the pancreas and pituitary. This explained the high Se level in kidney, the lesions occurring in cortex and medulla, the degenerative changes and necrosis found in the current study. The gross and histologic lesions reported in camel were comparable to those observed in lambs [48] and suggest that the heart, as target organ of selenium intoxication, failed, leading to pulmonary edema and hydrothorax [49]. 

The foot lesions with the necrosis of keratonocytes were comparable to those observed in alkali disease (chronic selenosis) in cattle [50] and horse [51], despite the lack of hooves in camel.

Selenium deficiencies in animal, including camel, can result also in damages to the liver, heart, kidney and skeletal muscles [27,52]. So, comparable necropsy lesions were reported on Se deficiency and toxicity. The lack or the excess of selenium seems to lead to similar cell damage.

According to the recommendations for beef cattle [53], the minimum level of selenium in the diet that causes chronic selenosis in most animal species is 4-5 mg/kg of dry matter (DM) and the minimum level needed to prevent deficiency is 0.02-0.05 mg/kg DM. In the experimental selenosis described by Seboussi *et al.* [10], the first symptoms appeared with a diet containing 2.5 mg/kg DM only. Chronic Se poisoning is not limited to grazing livestock and can occur from consumption of high Se intake in feed. For example, in the UAE, camels' owners supplement their animals to avoid deficiency with a commercial salt mixture and pharmaceutical form by drench or injection. However, no data on camel selenosis has been reported. The question of the *poisoning threshold* in camel has not been clearly determined. Oral ingestion of 1 to 2.2 mg of Se/kg life weight (LW) as sodium selenite has caused appreciable mortality in lambs up to 14 weeks of age [54], but individual susceptibility to selenosis could be highly variable. Tiwary *et al.* [48] did not observe lamb mortality with an oral sodium selenite up to 4 mg/kg LW. For other authors, the oral median lethal dose (LD50) of sodium selenite has been reported to be 1.9 ±1.2 mg of Se/kg LW [49,55,56,57,58]. A daily intake of 0.25 mg/kg LW is considered as toxic for sheep and cattle [57]. Selenium poisoning was observed with diet containing 44 mg/kg DM for horses and 11 mg/kg DM for pig [57]. Typical lesions of chronic selenium toxicosis were observed on young cattle receiving more than 5 mg/kg DM for 120 days [50].

These levels listed previously are higher than the dietary levels in the studies performed on camel [10,13], i.e., 0.051 to 0.095 mg/kg LW), which seems to show a high sensitivity of camel species to Se toxicosis. The levels of selenium requirement and toxicity could be very close. For example, in intoxicated lambs with 4 mg/kg LW under sodium selenite form (four times higher than the camels receiving 16 mg Se daily in the trial of Seboussi *et al.* [11], the serum Se increased up to 274 ng/mL only [48], compared to 767 ng/mL observed in camel [11]. 

After one month supplementation with 12 ppm Se in the diet, pregnant cattle showed Se values in serum above 420 ng/mL [58]. Higher values up to 1500 ng/mL were reported on large animals grazing on seleniferous pastures [51]. In lambs, with a diet containing 10 ppm of selenium [48], no toxicity was observed after one year and the selenium values reached 0.39 ppm in serum (390 ng/mL) after 12 weeks (comparatively to the results of Seboussi *et al.* [11]: after 90 days, 519 ± 97 ng/mL for groups receiving 3.5 ppm Se in the diet only).

## 10. Conclusions

The metabolism of selenium in camel is quite comparable to that of the other herbivores, with similar diseases in case of deficiency or toxicosis, comparable values in serum and organs and comparable mode of excretion. However, some specificities could be observed: the richness of camel milk in selenium, the role of fecal excretion in case of intoxication, the apparent sensitivity to toxicity, and the high concentration in blood with high Se supplementation. According to dietary Se supply and mean weight of the animal, selenosis appeared with 0.05 mg/kg LW Se supply only. Severe intoxication occurred with 16 mg Se supplementation, i.e., 0.10 mg/kg LW. These values were 5 times lower than those for sheep and cattle. Based on these results, it seems essential to limit Se supplementation in camel at 0.01-0.02 mg/kg LW, i.e., approximately 4-8 mg per day for adult animals or 0.5-1 ppm in the diet.
